# The contribution of pre-symptomatic infection to the transmission dynamics of COVID-2019

**DOI:** 10.12688/wellcomeopenres.15788.1

**Published:** 2020-04-01

**Authors:** Yang Liu, Centre for Mathematical Modelling of Infectious Diseases nCoV Working Group, Sebastian Funk, Stefan Flasche

**Affiliations:** 1 Centre for Mathematical Modelling of Infectious Diseases, Department of Infectious Disease Epidemiology, Faculty of Epidemiology and Population Health, London School of Hygiene & Tropical Medicine, London, London, WC1E 7HT, UK

**Keywords:** Pre-symptomatic transmission, Incubation period, Serial interval, COVID-19

## Abstract

**Background: **Pre-symptomatic transmission can be a key determinant of the effectiveness of containment and mitigation strategies for infectious diseases, particularly if interventions rely on syndromic case finding. For COVID-19, infections in the absence of apparent symptoms have been reported frequently alongside circumstantial evidence for asymptomatic or pre-symptomatic transmission. We estimated the potential contribution of pre-symptomatic cases to COVID-19 transmission.

**Methods:** Using the probability for symptom onset on a given day inferred from the incubation period, we attributed the serial interval reported from Shenzen, China, into likely pre-symptomatic and symptomatic transmission. We used the serial interval derived for cases isolated more than 6 days after symptom onset as the no active case finding scenario and the unrestricted serial interval as the active case finding scenario. We reported the estimate assuming no correlation between the incubation period and the serial interval alongside a range indicating alternative assumptions of positive and negative correlation.

**Results:** We estimated that 23% (range accounting for correlation: 12 – 28%) of transmissions in Shenzen may have originated from pre-symptomatic infections. Through accelerated case isolation following symptom onset, this percentage increased to 46% (21 – 46%), implying that about 35% of secondary infections among symptomatic cases have been prevented. These results were robust to using reported incubation periods and serial intervals from other settings.

**Conclusions: **Pre-symptomatic transmission may be essential to consider for containment and mitigation strategies for COVID-19.

## Introduction

Since its emergence in early December 2019, SARS-CoV-2 has spread rapidly despite multiple layers of interventions designed to prevent a pandemic ^1^. In less than two months the COVID-19 outbreak progressed from fewer than 300 cases across four countries to more than 100,000 cases in over 100 countries ^2, 3^. COVID-19 infection in the absence of clinical symptoms has been frequently described ^4– 7^ alongside anecdotal evidence for potential asymptomatic or pre-symptomatic transmission or transmission while symptoms were suppressed by fever-reducing and prescription-free medication ^8– 11^. In combination with highly non-specific and generally mild symptoms in most cases ^12^, pre-symptomatic transmission of COVID-19 would substantially hinder control and mitigation efforts, which typically focus on rapid syndromic identification and isolation of cases ^13, 14^. We thus estimated the possible extent of pre-symptomatic transmission to help inform infection control and mitigation plans.

## Data

Multiple estimates for the COVID-19 incubation period and serial interval have been reported to date. While estimates for the incubation period are relatively similar, the serial interval is dependent on the local context, in particular local contact patterns and the presence of interventions, active case finding with subsequent isolation, in particular, that may right-censor the serial interval. As our base case, we used the estimates provided by Bi *et al.* derived from 391 cases and their 1286 contacts as identified by the Shenzen Centre for Disease Control ^15^. This study currently includes the most extensive investigation of the infector-infectee transmission dynamics in a single setting.

## Methods

The probability of pre-symptomatic transmission can be expressed as the probability of onward transmission during one’s incubation period. Ideally, this probability could be estimated from individual-level observations on exposure, onset of symptoms and the transmission to others. However, such data remains largely unavailable and is inherently difficult to generate. Instead, we approximated this quantity from population estimates of the density distribution of the incubation period and the serial interval. We did so by first using the incubation period density distribution to calculate the probability of having developed symptoms on each day since infection and then using this to stratify the serial interval distribution, assumed to be a proxy for the generation time, into likely pre-symptomatic and symptomatic onward transmissions.

The observations of the incubation period and the serial interval may be correlated. In other words, individuals with a long incubation period may not have a serial interval of typical length but may be exactly those who have a long serial interval, or alternatively, exactly those who have a short serial interval. To account for this, we also calculated the proportion of pre-symptomatic transmission by randomly drawing from the incubation period and serial interval distributions and pairing samples not randomly, but aligning them either both in ascending or one in ascending and the other in descending order (rank ordering), before calculating the proportion of draws with a shorter serial interval than the incubation period. We report the estimates assuming no correlation accompanied by a range indicating estimates that assume positive and negative correlations.

**Figure 1.**
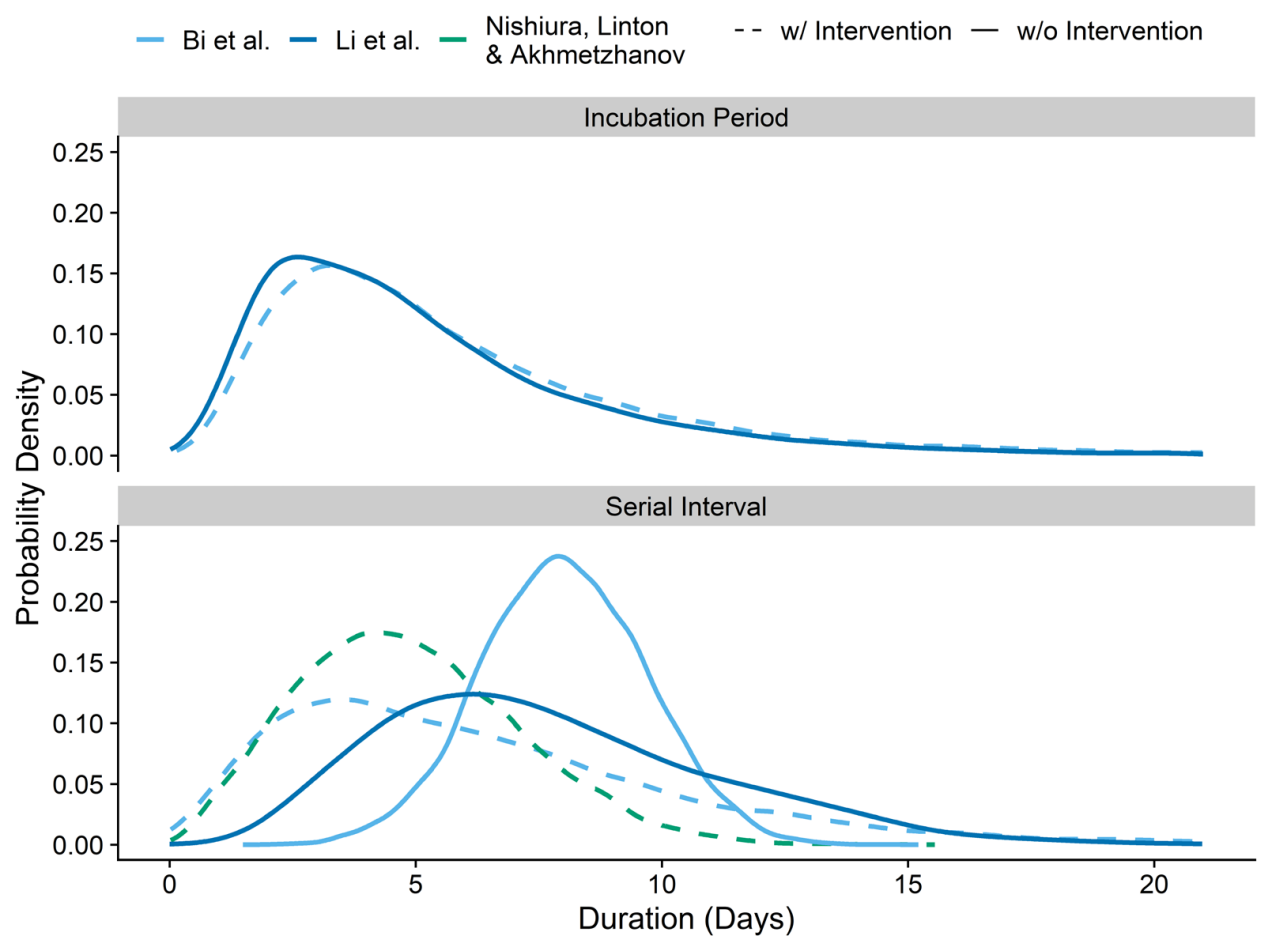
Probability density functions for the reported incubation period and serial intervals underlying our estimates.

We consider two scenarios: transmission with and transmission (largely) without active case finding to accelerate case isolation. For the no active case finding scenario we use the serial interval reported based on cases that were only isolated at least 6 days after their symptom onset; i.e. 8.0 days (95% empirical confidence interval (eCI): 4.7 – 11.3). For the active case finding scenario we used the overall serial interval; i.e. 6.3 days (95% eCI: 0.9 – 16.9) ( Figure 1). The incubation period in this study was on average 6.0 days (95% eCI: 1.3 – 17.2).

We also included sensitivity analyses. We used the reported mean incubation period (5.2 days (95% eCI: 1.1 – 14.3)) and serial interval (7.5 days (95% eCI: 2.5 – 15.3)) from the first 425 cases in Wuhan province as an alternative scenario for a setting with no or limited active case finding ^16^. Further, Nishiura *et al.* provide estimates of the serial interval from published reports across all affected countries, mostly including attempted rapid case finding and isolation ^17^. We use their mean serial interval estimates based on high certainty transmission pairs (4.8 days (95% eCI: 1.1 – 9.6)) in combination with either the incubation time reported by Bi *et al.* ^15^ or Li *et al.* ^16^. We implemented all density distributions to match reported estimates and their distributions ^18^.

We then calculated the effectiveness of case isolation in reducing the number of secondary cases during the symptomatic period ( *ϕ*) Let *x* be the proportion of pre-symptomatic transmission in the absence of active case finding, and *y* the same proportion in the presence of active case finding. Then, *y* can be expressed as a function of *x* while accounting for a reduction *ϕ* in the symptomatic cases:

$$y\;=\;\frac{x}{x\;+\;\phi \;(1\;–\;x)}$$

Solving for *ϕ*:

$$\phi \;=\;\frac{\frac{x}{y}\;–x}{1\;–\;x}\;=\;\frac{x(1\;–\;y)}{y(1\;–\;x)}\;$$

All analyses were done in R 3.6.3 ^19^; codes and data are available online ^18^.

## Results

**Figure 2.**
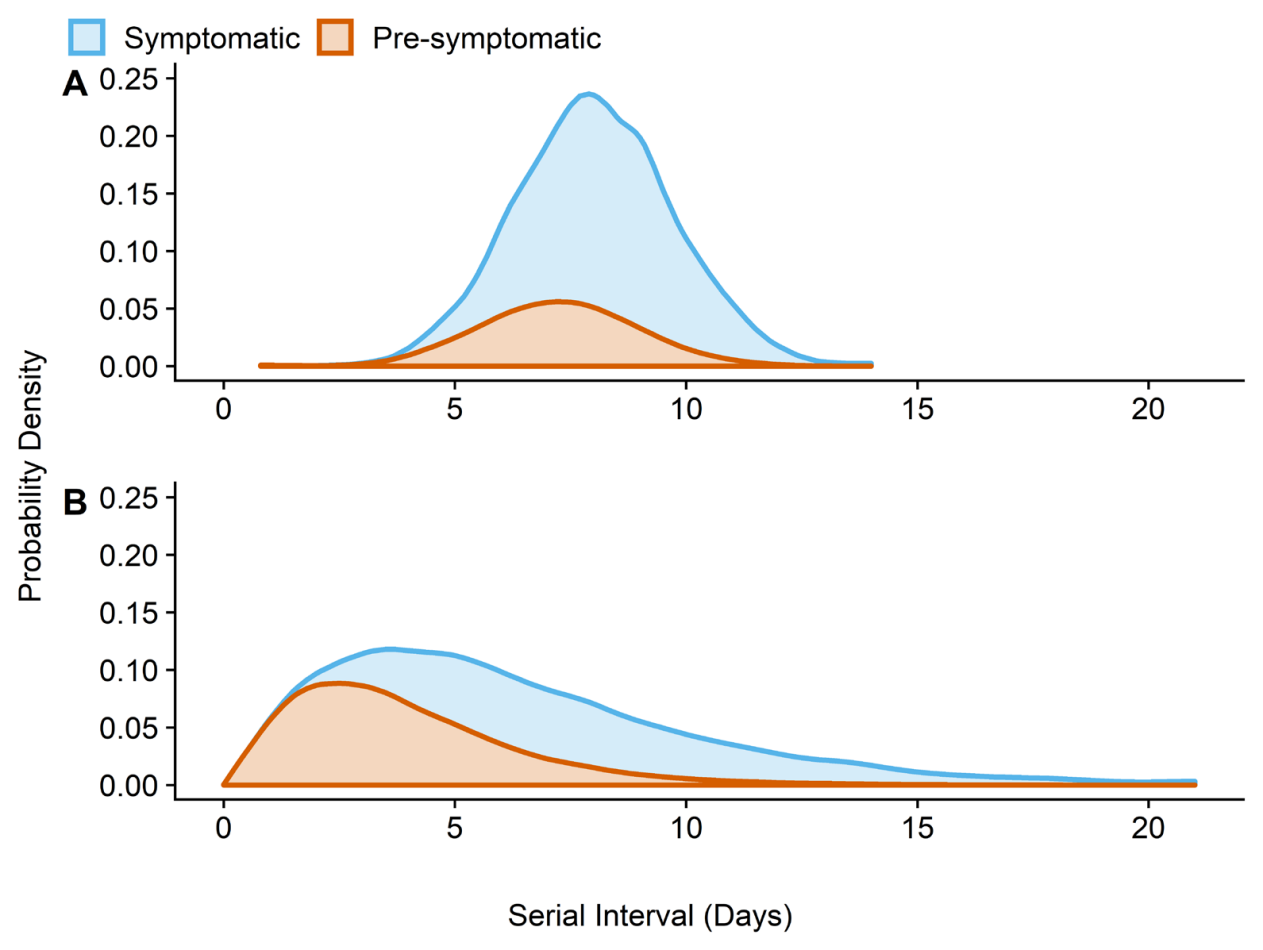
Estimated attribution of the serial interval into pre-symptomatic transmission and symptomatic transmission for ( **A**) no active case finding and ( **B**) active case finding and accelerated case isolation. These estimates assume uncorrelated incubation period and serial interval estimates from Shenzen.

In the scenario of no active case finding and isolation before six days after symptom onset, and assuming uncorrelated serial interval and incubation period distributions, we estimate that 23% (range accounting for correlation: 12 – 28%) of onward transmissions in Shenzen have occurred during the pre-symptomatic period ( Figure 2 and Table 1).

**Table 1.** Overview of scenarios tested and the corresponding estimates for the percentage of transmission that may be attributable to pre-symptomatic infections.

Scenario	Active case finding	Serial interval (95% eCI) *	Incubation period (95% eCI) *	Estimated percentage of pre-symptomatic transmission if the incubation period and the serial interval are		
				Uncorrelated	Fully correlated	Fully anti-correlated
Main analyses	No	Bi *et al*.	Bi *et al*.	23%	12%	28%
		8.0 (4.7 – 11.3)	6.0 (1.3 – 17.2)			
	Yes	Bi *et al*.	Bi *et al*.	46%	21%	46%
		6.3 (0.9 – 16.9)	6.0 (1.3 – 17.2)			
Sensitivity analyses	No	Li *et al*.	Li *et al*.	27%	1%	32%
		7.5 (2.5 – 15.3)	5.2 (1.1 – 14.3)			
	Yes	Nishiura *et al*.	Li *et al*.	48%	38%	47%
		4.8 (1.1 – 9.6)	5.2 (1.1 – 14.3)			
	Yes	Nishiura *et al*.	Bi *et al*.	53%	66%	51%
		4.8 (1.1 – 9.6)	6.0 (1.6 – 14.0)			

* A summary table of the parameters used and the underlying assumptions regarding the probability distributions used is publicly available ^18^. The uncertainty ranges above are those of the simulated distributions of serial interval and incubation periods (instead of the mean or median of these parameters), and thus may be different from what is reported in the original articles. Note that serial intervals sampled for with and without active case finding scenarios were independent, i.e., based on separate sets of parameters.

Active case finding and subsequent accelerated isolation right censors the serial interval and thereby reduces the number of symptomatic onward transmissions. In Shenzen, the presence of active case finding increased the percentage pre-symptomatic among all transmissions to 46% (21 – 46%). Therefore, case isolation in Shenzen has on average reduced the number of secondary cases during the symptomatic period by 35% (42 – 59%) and hence the total number of secondary cases by 27% (32 – 45%).

Both the sensitivity analyses for the scenario of limited to no active case finding and for the scenario of real-world effects of active case finding and isolation during the containment phase of the outbreak response are similar to the estimates in our main analysis ( Table 1).

## Conclusion

We estimated that without active case finding to accelerate isolation in Shenzen 12 to 28% of transmissions occurred during the pre-symptomatic period. We further estimated that active case finding reduced the number of secondary infections after symptom onset and thereby increased the percentage of pre-symptomatic transmissions to 21 to 46%. This implies that in Shenzen accelerated case isolation has prevented 35 – 60% of secondary cases among symptomatic infectees. These results were similar when alternative settings with and without accelerated case isolation were considered.

**Figure 3.**
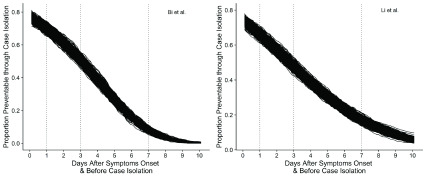
Proportion of potentially preventable onward transmission by days after symptoms onset. These estimates are based on parameters from scenarios with no public health interventions.

In addition to the mostly-mild and non-specific symptoms of COVID-19, a high contribution of pre-symptomatic cases to the transmission dynamics of COVID-19 could be part of the reason why controlling COVID-19 has been challenging and seems to have failed in many countries ^13^. It implies that control and mitigation of COVID-19 may be substantially improved if also targeting pre-symptomatic infections. This may be achieved via social distancing interventions that do not rely on the identification of symptoms. Having 10 – 30% pre-symptomatic transmission also suggests that immediate isolation at symptom onset can at best prevent 70–90% of cases. However, immediate case isolation on the day of symptoms onset is unlikely. We also find that, based on parameters estimated under no active case finding scenarios, case isolation on days 1, 3 or 7 after symptom onset reduces the proportion of or preventable onward transmissions by syndromic case finding and isolation to approximately 60%, 50% and less than 20%, respectively ( Figure 3). We find that in Shenzhen syndromic identification and isolation has prevented 35 – 60% of secondary cases from symptomatic infectees, which is in line with previous model-based estimates ^14^.

Even for well-studied pathogens like influenza, there is a substantial amount of uncertainty in the contribution of asymptomatic and pre-symptomatic infections to the transmission dynamics. For influenza, 10 – 20% of infections have been found to be asymptomatic but shedding, although at lower viral loads ^20, 21^. For SARS, 7.5% of exposed and seroconverted health care workers were found to not have displayed symptoms although it is unclear if those were also effectively transmitting ^22^. For COVID-19 the proportion of pre-symptomatic or asymptomatic infections is likely much higher. During an outbreak aboard a cruise ship, 50% of passengers who tested positive were asymptomatic at sample collection and therefore may or may not have developed symptoms at some point later on ^6^. Whether asymptomatic carriers are also infectious has not yet been clearly established. Our work is based on observations of eventually symptomatic cases and hence we implicitly assume that only those who will eventually become symptomatic can effectively transmit. If there is an additional role for asymptomatic individuals in the transmission of COVID-19 this will further decrease the proportion of secondary infections attributable to symptomatic cases. Hence, our estimate that among those COIVD-19 cases who eventually will develop symptoms the pre-symptomatics substantially contribute to transmission is a conservative approximation for the contribution of individuals without symptoms to COVID-19 transmission.

A key limitation for this study is that we had to use population-level estimates rather than individual-level estimates for the incubation period and the serial interval. We address biases potentially arising from the correlation of the two estimates on the individual level by estimating the sensitivity of estimates to the assumption of positive or negative correlation. Additionally, our estimates are in essence estimating the proportion of pre-symptomatic transmission from the amount of overlap of the incubation period distribution and the serial interval distribution. If the heterogeneity observed in either distribution was originated in measurement bias rather than in actual between individual heterogeneity of the infection process, then we would overestimate the role of pre-symptomatic transmission. In our sensitivity analyses, we find similar estimates for other settings indicating that the observed heterogeneity in Shenzhen is unlikely to be purely an observation artefact. However, our study supports the World Health Organization’s prioritisation for epidemiological studies, ideally individual-level observations, to further enhance the evidence base around the role of pre-symptomatic transmission for COVID-19 ^23^.

In summary, we find that pre-symptomatic transmission may play a key role in the epidemiology of COVID-19. Given the relevance of pre-symptomatic transmission for control and mitigation efforts, additional studies are urgently needed to reduce the uncertainty in the role of pre-symptomatic transmission.

## Data availability

### Source data

This project does not involve any primary data collection. All data used are based on references 15, 16, and 17. More specifically:

nCoV_Incubation.csv (Digitized distribution of incubation period from 16)

nCoV_Serial.csv (Digitised distribution of serial interval from 16)

COVID-19 Serial Interval.csv (Detailed information on parameters used to reconstruct distributions of serial interval and incubation period from 15, 16, and 17).

### Underlying data

Zenodo: yangclaraliu/2019nCoV_proportion_asym: WOR Submission. https://doi.org/10.5281/zenodo.3709942 ^18^.

This project contains the following underlying data:

- COVID-19 Serial Interval.csv (Spreadsheet of extracted source data)

Data are available under the terms of the Creative Commons Zero "No rights reserved" data waiver (CC0 1.0 Public domain dedication).

## Software availability

Replication code is available from:

https://github.com/yangclaraliu/2019nCoV_proportion_asym

Archived source code at the time of publication: https://doi.org/10.5281/zenodo.3709942 ^18^.

License: CC0 1.0 Universal
