# Supplementary material for: The contribution of pre-symptomatic infection to the transmission dynamics of COVID-2019
Source: Wellcome Open Res. 2020 Apr 1;5:58. [Version 1] doi: 10.12688/wellcomeopenres.15788.1 (PMC7324944; doi:10.12688/wellcomeopenres.15788.1)
Supplement: Peer review history

# Peer review: Reviewer response for version 1

**DOI:** 10.21956/wellcomeopenres.17314.r39156

**Published:** 2020-07-06

**Authors:** Elizabeth Lee

**Affiliations:** 1 Department of Epidemiology, Johns Hopkins Bloomberg School of Public Health, Baltimore, MD, USA

This report by Liu *et al.* is a clean and straightforward analysis that brings together early data on the incubation period and serial interval of COVID-19 to provide important insight into the contributions of pre-symptomatic individuals to onward disease transmission. The authors' analysis is strengthened by the inclusion of sensitivity analysis on assumptions about the incubation period and serial interval as well as scenarios that account for the impact of active case finding on the relative contribution of pre-symptomatic infections to onward transmission.

 Comments:

- I would like a more explicit description of how the pre-symptomatic transmission percentages were estimated. As is, I'm not sure why there are only point estimates presented in Table 1, unless only a single set of draws were performed for each category.
- Can the authors comment on the sufficiency of follow up time for the studies that informed the serial interval estimates? Are the authors reasonably confident that there is limited right censoring for individuals that were non-symptomatic and positive at identification (i.e., to see whether they may have instead been pre-symptomatic). This seems an important consideration that would bolster the validity of the authors' estimates.
- Since this paper was originally posted, I am wondering whether there have been additional estimates of the serial interval under conditions of active case finding. Presumably locations outside of China may have had time to prepare their surveillance systems so that serial interval estimates would directly reflect scenarios with active case finding (as opposed to making a cutoff assumption).

Is the work clearly and accurately presented and does it cite the current literature?

Yes

If applicable, is the statistical analysis and its interpretation appropriate?

Yes

Are all the source data underlying the results available to ensure full reproducibility?

Yes

Is the study design appropriate and is the work technically sound?

Yes

Are the conclusions drawn adequately supported by the results?

Yes

Are sufficient details of methods and analysis provided to allow replication by others?

Partly

Reviewer Expertise:

infectious disease dynamics

I confirm that I have read this submission and believe that I have an appropriate level of expertise to confirm that it is of an acceptable scientific standard.

# Peer review: Reviewer response for version 1

**DOI:** 10.21956/wellcomeopenres.17314.r39157

**Published:** 2020-06-29

**Authors:** Andrei R. Akhmetzhanov

**Affiliations:** 1 Graduate School of Medicine, Hokkaido University, Sapporo, Japan

I have found the paper of Liu *et al. *to be rather interesting and enjoyed reading it. The analysis is well-designed and the main formula in the Results section is quite elegant. I also think that the results lead to important conclusions. 

 I have only few remarks listed below.

 Remarks:

- Table 1: It would interesting if the authors could give some insight on “fully anti-correlated” SI and incubation period. Under which circumstances, may this happen? I could imagine uncorrelated and fully correlated cases, probably, partially anti-correlated, but it was difficult for me to imagine the setting of fully anti-correlated cases.

Typos, etc:

- Page 6, sixth line from above: probably, the authors could specify that it was the Diamond Princess cruise ship docked in Japan.
- Figure 3: I would recommend to make the lines grey and a bit transparent, so the reader would be able to distinguish between individual lines.
- Github repo (very optional): the author could improve their README.md file, so to make it as a graphical abstract of their paper. This may help to ease the access to the repo for the readers.

Is the work clearly and accurately presented and does it cite the current literature?

Yes

If applicable, is the statistical analysis and its interpretation appropriate?

Yes

Are all the source data underlying the results available to ensure full reproducibility?

Yes

Is the study design appropriate and is the work technically sound?

Yes

Are the conclusions drawn adequately supported by the results?

Yes

Are sufficient details of methods and analysis provided to allow replication by others?

Yes

Reviewer Expertise:

Infectious disease modeling

I confirm that I have read this submission and believe that I have an appropriate level of expertise to confirm that it is of an acceptable scientific standard.

# Peer review: Reviewer response for version 1

**DOI:** 10.21956/wellcomeopenres.17314.r38330

**Published:** 2020-04-08

**Authors:** Benjamin J. Cowling

**Affiliations:** 1 WHO Collaborating Centre for Infectious Disease Epidemiology and Control, School of Public Health, Li Ka Shing Faculty of Medicine, The University of Hong Kong (HKU), Hong Kong, China

This is a nice simple analysis of the incubation period distribution and the serial interval distribution, showing that substantial pre-symptomatic transmission has likely been occurring.

 There is fairly good consistency in published estimates of the incubation period distribution, but relatively less consistency in published estimates of the serial interval distribution. Authors deal with this variability/uncertainty through sensitivity analyses.

**Minor comments:**

1. Figure 3 is difficult to read, can you use semitransparency to distinguish the density of simulated lines?
2. Do you have any estimate of how early people might become contagious before symptom onset? In Figure 3 you show infectivity is persisting for up to a week after onset, but how many days before onset did infectiousness start?

Is the work clearly and accurately presented and does it cite the current literature?

Yes

If applicable, is the statistical analysis and its interpretation appropriate?

Yes

Are all the source data underlying the results available to ensure full reproducibility?

Yes

Is the study design appropriate and is the work technically sound?

Yes

Are the conclusions drawn adequately supported by the results?

Yes

Are sufficient details of methods and analysis provided to allow replication by others?

Yes

Reviewer Expertise:

Infectious disease epidemiology

I confirm that I have read this submission and believe that I have an appropriate level of expertise to confirm that it is of an acceptable scientific standard.
